# Novel immunoassay for TSH measurement in rats

**DOI:** 10.1590/2359-3997000000293

**Published:** 2017-09-04

**Authors:** Thalita G. Alves, Maria Clara de C. Melo, Teresa S. Kasamatsu, Kelen C. Oliveira, Janaina Sena de Souza, Rodrigo Rodrigues da Conceição, Gisele Giannocco, Magnus R. Dias-da-Silva, Maria Izabel Chiamolera, José Gilberto Vieira

**Affiliations:** 1 Divisão de Endocrinologia Departamento de Medicina Universidade Federal de São Paulo São Paulo SP Brasil Laboratório de Endocrinologia Molecular e Translacional, Divisão de Endocrinologia, Departamento de Medicina, Universidade Federal de São Paulo (Unifesp), São Paulo, SP, Brasil; 2 Departamento de Bioquímica Escola Paulista de Medicina Unifesp São Paulo SP Brasil Divisão de Biologia Molecular, Departamento de Bioquímica, Escola Paulista de Medicina, Unifesp, São Paulo, SP, Brasil; 3 Grupo Fleury São Paulo SP Brasil Grupo Fleury, São Paulo, SP, Brasil; 4 Departamento de Ciências Biológicas Unifesp Diadema SP Brasil Departamento de Ciências Biológicas, Unifesp, Diadema, SP, Brasil

**Keywords:** Immunoassay, rats, TSH

## Abstract

Measuring thyroid hormones is an important aspect for the study of metabolism and for monitoring diseases in both human and animal models. The traditional method for hormone measurement in rats is the radioimmunoassay (RIA). However, the RIA is associated with some practical disadvantages, including the use of radioactive material, the need for specialized equipment and expert staff, the short shelf-life of kits according to the half-life of the radioisotope and high costs. The objective of this study was to develop a new cost-effective method for measuring TSH levels in rats that avoids the use of radioactive material. We developed an in-house competitive immunoassay using a reference standard, polyclonal antibody produced in rabbits and biotinylated antigen. This method was tested in 64 Wistar rats that were divided into a control group (n = 41) and a group with hypothyroidism (n = 23). Our assay demonstrated an analytical sensitivity of 0.24 ng/mL (n = 12) and an intra-assay coefficient of variation (CV) of 8.9% for sera with TSH levels of 1.5 ng/mL and 13.2% for sera with TSH levels of 17.5 ng/mL (n = 14). The inter-assay CV was 13.5% for sera with TSH levels of 1.4 ng/mL and 14.5% for TSH levels of 18.2 ng/mL (n = 5). The analysis of mean TSH levels in control rats (5.06 ± 0.5701) and hypothyroid rats (51.09 ± 5.136) revealed a statistically significant difference (p < 0.001) between the groups. This method showed good sensitivity, can be automated and is low-cost compared with RIA. Our method offers a viable alternative for TSH measurement in rats.

## INTRODUCTION

Hormone measurement is an important component for the study of metabolism, analysis of diseases and as a parameter of health assessments. Of the different hormone assays, immunological assays have become standard due to several advantages: they offer great specificity and potentially high sensitivity and present with ease of use and wide-ranging applications (1). Immunological assays are largely used for measuring biologically active compounds present in low concentrations, such as hormones, proteins, drugs and microorganisms. In this context, the most commonly used techniques are radioactively labeled competitive and non-competitive immunoassays (RIA, IRMA), which are highly sensitive and precise but are also associated with several disadvantages. Among the drawbacks, the use of radioactive tracers is of most concern because they present a health hazard, require special attention for handling, well-trained staff and well-defined waste storage. Moreover, because radioisotopes have a limited half-life, kits have limited shelf lives, and radioactive counting can be time-consuming and requires expensive instrumentation. Because of these issues, alternative immunoassays (competitive or non-competitive) have been developed using alternative labels such as enzymes (EIA, ELISA), luminescent compounds, fluorescent probes (IFMA, FIA) and metals. Assays that use alternative tracers offer similar or better sensitivity than that of IRMA and RIA. Importantly, avoidance of radiation is a major advantage.

Several immunoassays for measuring hormones have been described, and the most commonly used technique for the measurement of rat serum is the radioimmunoassay (RIA), which is associated with the disadvantages described above (2). In addition, commercial kits are costly, and their shelf life depends on the half-life of the radioisotope. Therefore, development of new assays that do not use radioisotopes is needed.

## MATERIALS AND METHODS

### Assay development

We developed an in-house competitive immunoassay using rat TSH extracted from pituitary gland as a standard, polyclonal antibody produced in rabbits against rat TSH and biotinylated TSH from the same source (National Institute of Diabetes & Digestive & Kidney Diseases, NIDDK). Rat thyroid-stimulating hormone was prepared at a concentration of 50 µg/mL, after which it was biotinylated using the *EZ-link-Sulfo-NHS-LC-Biotin* kit according to the manufacturer’s protocol (Thermo Scientific). A six-value curve was constructed from serial dilution of standard rat TSH in bovine fetal serum at the following concentrations: 100, 25, 6.25, 1.56, 0.39 and 0 ng/mL. The standards, rat sera used as controls and test samples were incubated with a rabbit polyclonal antibody provided by NIDDK. After 24 h of incubation at 4°C, biotinylated TSH was added to tubes at a dilution of 1:1000. After another 24 h incubation at 4°C, samples were transferred to a microtiter plate (Fluoronunc, Nunc, Roskilde, Denmark) that was previously adsorbed with an anti-rabbit IgG monoclonal antibody (D_4_P_4_) produced by our laboratory (monoclonal antibody, 2 µg/well). After a 4-h incubation at room temperature and subsequent washing (Tris-HCl 50 mM with 0.5% BSA and 0.1% gamma-globulin), a solution of streptavidin-europium was added (200 µL/well). Finally, after incubation for 30 min at room temperature, plate was washed 12 times with buffer, and an enhancement solution was added (200 µL/well, Delfia Enhancement Solution, Perkin-Elmer, Turku, Finland). The plate was then read by time-synchronized fluorometry in a Victor3 time-resolved fluorometer (Perkin-Elmer, Turku, Finland). The amount of analyte was calculated based on the standard curve, and the results are expressed in ng/mL.

### Animals

To test our method, we measured the serum TSH levels of 64 adult male Wistar rats (200-250 g) from the animal facility of the Institute of Pharmacology of Unifesp (Infar). Rats were kept under standard conditions for temperature (25 ± 1°C) and light/dark cycle (12/12 hours per day). Drinking water and food were provided *ad libitum.* Animals were divided into two experimental groups: a control group and a group with hypothyroidism. The hypothyroidism group consisted of 23 animals that were subjected to total thyroidectomy followed by methimazole treatment for 20 days. The control group consisted of 41 rats subjected to sham surgery.

### Statistical analysis

All results are presented as mean values ± standard error of the mean. Statistical analysis was performed in Graph Pad Prism^®^ version 5 software for Windows. First, the Shapiro-Wilk normality test was performed to verify normality of the distribution of the two sample groups. Non-parametric distribution was observed in the two groups, and thus a Mann-Whitney test was used to compare control and hypothyroidism groups. For all analyses, a significance threshold of p < 0.05 was assumed.

### Ethics

All procedures were performed in accordance with the Brazilian College of Animal Experimentation and were approved by the Bioethical Commission of the Universidade Federal de São Paulo (Unifesp) (protocol number 5097101316).

## RESULTS

Analytical sensitivity was determined using TSH stripped fetal bovine serum (FBS). FBS was measured multiple times, from which we calculated mean and standard deviation. Sensitivity was defined by the concentration corresponding to the mean - 2 standard deviations of the latter measurements. A 95% confidence interval was obtained, and the limit was defined by doubling the standard deviation. The new assay showed sensitivity of 0.24 ng/mL (n = 12). Intra-assay CV was 8.9% for sera with TSH levels of 1.5 ng/mL and 13.2% for sera with TSH levels of 17.5 ng/mL (n = 14). Inter-assay CV was 13.5% for sera with TSH levels of 1.4 ng/mL and 14.5% for sera with TSH levels of 18.2 ng/mL (n = 5). The values in the control rats ranged between 0.81 and 19.50 ng/mL, whereas the values in hypothyroid rats ranged between 15 and > 100 ng/mL.

Linearity of our assay was tested by serial dilution of a hypothyroid rat serum (36 ng/mL), which produced a value of R^2^ = 0.97. The analysis of mean TSH obtained from control rat sera (5.06 ± 0.5701) and hypothyroid rat sera (51.09 ± 5.136) showed statistically significant differences (p < 0.001) between the groups.

To verify cross-reaction with the antibody used in our test, sera from different species were tested: FBS, mouse, rabbit, horse and primate. Rabbit serum interferes significantly in the assay, although sheep and horse also have some interference, generating a lower reading signal. Then, we used standard TSH to create curves using these different matrices, to identify if assay sensitivity could be improved. Curves in FBS and in buffer with 5% BSA are the most sensitive, although FBS remains the best matrix since, in buffer, the curve is slightly shifted to the right, indicating lower analytical sensitivity. Horse and sheep sera are much less sensitive (data not shown).

As a recovery test we used three sera samples with different TSH concentrations and added 50 ng of the standard in the two matrices.

**Figure 1:**
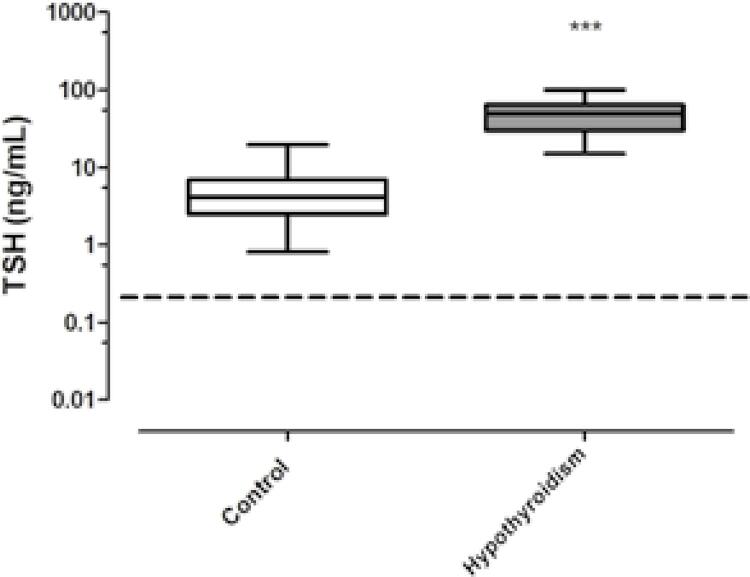
Mean TSH levels (± SEM) measured using a novel fluoroimmunoassay in 41 control and 23 rats with hypothyroidism. Dashed line indicates the lower limit of detection of the assay.

## DISCUSSION

Measurement of TSH levels is useful for clinical evaluation of human patients, as small changes in free thyroid hormone levels lead to greater changes in TSH levels. Serum TSH is considered the single most reliable diagnostic test for all common forms of hypothyroidism and hyperthyroidism. Animal research has played a vital part in medical and biomedical research and has led to important scientific discoveries in many fields, including endocrinology (3). Therefore, more sensitive and specific tests in animal field could increase the frequency of testing and, consequently, detection of thyroid conditions, with the possibility of detecting degrees of thyroid dysfunction in research animals.

In this study, we developed a new solid-phase fluorometric immunoassay for measuring TSH in rats that does not use radioactive material. The lack of radioactive material offers a considerable advantage because handling radioactive material presents a health hazard for researchers, and the assay shelf life is not constrained by the half-life of the radioisotope. Our method shows good sensitivity, can be automated, and with its low operational cost offers considerable advantages compared with traditional RIA methods. Additionally, as NIDDK reagents are the same as those used in the traditional RIA, following recommendations in ATA Guidelines for thyroid hormone investigation in rodents and cell models (2), this assay can be compared with available data in the literature.

However, our method has some limitations. In recovery test, all samples recovered above the expected (data not shown). One reason that should be taken in consideration is the fact that the standard originates from rat pituitary. Unfortunately, there is no source of rat serum TSH to use as the standard for assembling the immunoassay, forcing us to use pituitary TSH. The antibody used in the assay was also produced against pituitary TSH, and, probably, recognizing it more specifically and with greater affinity than serum TSH. For this reason, the assay shows high recovery. This is a limitation of experimental design, since all the assay reagents are derived from pituitary TSH, that has a different immunogenic structure than rat serum TSH.

More studies measuring TSH levels in rats with hyperthyroidism should be conducted. Nevertheless, the method described here offers an effective alternative for TSH measurement in rats.

Acknowledgements: the authors are grateful to Angela Faria, Gilberto Furusawa, Ilda Kunii and Teresa Kasamatsu for administrative and technical support. We also thank Dr. A. F. Parlow for his contributions to the hormone measurements over the course of the several studies of our group and for providing reliable reagents.

## References

[1] Hemmilä I. Fluoroimmunoassays and immunofluorometric assays. Clin Chem. 1985;31(3):359-70. 3882272

[2] Bianco AC, Anderson G, Forrest D, Galton VA, Gereben B, Kim BW, et al. American Thyroid Association Guide to investigating thyroid hormone economy and action in rodent and cell models. Thyroid. 2014;24(1):88-168. 10.1089/thy.2013.0109 PMC3887458 24001133

[3] Bahn Chair RS, Burch HB, Cooper DS, Garber JR, Greenlee MC, Klein I, et al. Hyperthyroidism and other causes of thyrotoxicosis: management guidelines of the American Thyroid Association and American Association of Clinical Endocrinologists. Thyroid. 2011;21(6):593-646. 10.1089/thy.2010.0417 21510801

